# Osteogenic mechanism of deciduous teeth periodontal ligament stem cells in inflammatory environment

**DOI:** 10.1590/1414-431X2024e13606

**Published:** 2024-10-07

**Authors:** Jin-yi Li, Shan-shan Dai, Zheng-yang Li, Qing-yu Guo, Fei Liu

**Affiliations:** 1 Key Laboratory of Shaanxi Province for Craniofacial Precision Medicine Research, College of Stomatology, Xi'an Jiaotong University, Xi'an, China; 2 Department of Pediatric Dentistry, College of Stomatology, Xi'an Jiaotong University, Xi'an, China

**Keywords:** Apical periodontitis, Deciduous teeth, Inflammation, Osteogenesis, PI3K-AKT signaling pathway

## Abstract

This study aimed to illustrate the biological behavior and changes in cell function during the progression of apical periodontitis in deciduous teeth and to explore the underlying molecular mechanism. Deciduous teeth periodontal ligament stem cells (DePDLSCs) were derived and their identity was confirmed. The viability, inflammation, and osteogenic ability of cells were tested by exposing them to various concentrations of lipopolysaccharide (LPS) (0-100 μg/mL) using the cell counting kit-8 (CCK-8) assay, reverse transcription polymerase chain reaction (real-time PCR), alkaline phosphatase (ALP) staining, and ALP activity assay. In addition, osteogenic-induced cells with and without 10 μg/mL LPS were harvested for high-throughput sequencing. Based on sequencing data, proinflammatory factors and ALP expression were measured after interference with the PI3K-AKT signaling pathway activator, 740Y-P. LPS biphasically affected the proliferation and osteogenesis of DePDLSCs. Low concentrations of LPS showed stimulatory effects, whereas inhibitory effects were observed at high concentrations. Sequencing analysis showed that the PI3K-AKT signaling pathway was significantly downregulated when DePDLSCs were treated with 10 μg/mL LPS. The LPS-induced inflammation and osteogenesis inhibition of DePDLSCs were partially rescued by 740Y-P treatment. In conclusion, LPS affected DePDLSCs proliferation and osteogenesis in a biphasic manner. Moderate activation of PI3K-AKT signaling pathway was beneficial for osteogenic differentiation and anti-inflammatory effect in DePDLSCs. This research may provide etiological probes for apical periodontitis and its treatment.

## Introduction

The primary dentition is crucial for children's nutritional intake, digestion, craniomaxillofacial development, facial aesthetics, and pronunciation. However, according to The Fourth National Oral Health Epidemiological Survey, the burden of dental caries among children in China is high and the treatment rate is very low (1). Owing to its histological and anatomical features, caries in the deciduous tooth can easily progress to irreversible pulpitis or apical periodontitis, resulting in pain, maxillofacial infection, and premature tooth loss (2). In addition to abnormal masticatory habits, pronunciation problems, loss of arch length, and altered eruption of successors, the loss of deciduous molars may result in undernutrition and impaired development (3). Consequently, the pediatric dentist should strive to prolong the longevity of the afflicted tooth.

Root canal therapy (RCT) is the final conservative option for deciduous teeth diagnosed with irreversible pulpitis or apical periodontitis without severe root resorption. A few clinical studies, most of which were brief follow-up investigations of no more than 2 years, have surveyed the efficacy of RCT in deciduous teeth and revealed a success rate ranging from 50 to 81.3% (4,5). In addition, radiographic failure appears in more than half of the RCT-treated deciduous teeth, which exfoliate prematurely (4). The failure of RCT-treated deciduous teeth is mostly attributed to advanced root resorption, and the resorption speed is unpredictable. Apart from several clinical factors (6,7), the complex apical microenvironment of deciduous teeth may lead to an uncertain prognosis.

The complexity of apical periodontitis in deciduous teeth is due to the co-existence of bacterial-derived inflammation and physiological root resorption. Endodontic infections of apical periodontitis are predominately caused by Gram-negative bacteria, which trigger local immune responses around the apical tissues and cause bone resorption (8). Lipopolysaccharide (LPS), a component of Gram-negative bacteria, can induce inflammation by activating the secretion of numerous cytokines such as interleukin (IL)-6, IL-1β, and tumor necrosis factor (TNF)-α (9). LPS accumulation is closely related to the radiographic and clinical symptoms of apical periodontitis (10). In addition, *Escherichia coli*, one of the important pathogens of apical periodontitis, is frequently detected in the infected root canals of deciduous teeth and has been extensively applied for apical periodontitis modeling (11,12). In physiological conditions, periodontal ligament stem cells (PDLSCs) play an important role in root resorption of deciduous teeth and subsequent germ eruption (13). Deciduous teeth periodontal ligament stem cells (DePDLSCs) were firstly isolated by Ji et al. (14) and manifested mesenchymal stem cell-like properties; however, DePDLSCs differ from stem cells of permanent teeth concerning developmental microenvironment, proliferation, differentiation capacity, and gene expression (13,15). Inflammation might change the characteristics of DePDLSCs, resulting in homeostatic imbalance of the root tissue and ultimately root resorption, yet the molecular mechanisms remain unknown.

Since there are few relevant studies, this study performed high-throughput sequencing based on an *in vitro* DePDLSCs inflammation model to illustrate how apical periodontitis affected biological behavior and cell function and to shed light on potential new treatments.

## Material and Methods

### Cell culture

The study protocol was approved by the Ethics Committee of College of Stomatology, Xi'an Jiaotong University (xjkq11[2021] No. 09). The guardians of children provided consent. DePDLSCs were isolated from retained deciduous teeth with root resorption of more than two-thirds and teeth undergoing extraction for dental trauma or orthodontic purposes. As described before (14), the extracted teeth were rinsed with phosphate buffered saline (PBS, Boster, China) containing antibiotics(100 U/mL penicillin and 100 mg/mL streptomycin, Sigma, USA), and periodontal ligament (PDL) tissue from the middle part of the root was digested with type I collagenase (3 mg/mL, Sigma) and dispase (4 mg/mL, Sigma). After digestion, tissue pieces were seeded onto 25-cm^2^ flasks and incubated in α-minimum medium (α-MEM, Hyclone, USA) supplemented with 20% fetal bovine serum (FBS, Biological Industries, Israel). Adherent cells reached approximately 70% confluence 14 days after primary culture. They were further purified and serially subcultured. Cells from passages 2-4 were used in subsequent assays.

### Flow cytometry analysis

After washing with cold PBS (0.1% bovine serum albumin added), 1×10^6^ cells were collected and incubated with PE-labeled antibodies (STRO-1, CD29, CD34, and CD45) and FITC-labeled antibody CD105 in the dark at 4°C for 30 min. The antibody STRO-1 was bought from Abcam (UK) and the others were obtained from BD Pharmingen (USA). After washing twice with cold PBS, the cell suspension was analyzed using a flow cytometer (BD FACSCelesta™, BD Biosciences, USA) for mesenchymal stem cell identification.

### Multipotentiality of DePDLSCs

As soon as the confluence of seeded cells reached 80%, the osteogenic medium containing 100 nM dexamethasone, 50 μg/mL ascorbic acid, and 10 mM β-glycerophosphate was added and replaced every 3 days. Three weeks later, an Alizarin red staining assay was performed. For adipocyte induction, a standard adipocyte medium (Cyagen, China) was used according to the manufacturer's instructions. Furthermore, lipid droplets were detected using Oil Red O staining after 4 weeks of differentiation. Briefly, cells were fixed with 4% paraformaldehyde for 20 min after washing with PBS, and rinsed twice in distilled water. Plates were stained with Alizarin red staining solution (Solarbio, China) or Oil Red O staining solution (Cyagen) for 30 min. Positively stained mineral deposits and oil droplets were observed and captured by inverted microscope (Olympus, Japan).

### Cell proliferation assay

DePDLSC seeded onto 96-well plates (1000 cells per well) were cultivated in a medium containing LPS (*E. coli* 05*5:B5*, Sigma) at a series of concentrations (0, 0.05, 0.1, 1, 10, 50, and 100 μg/mL) or with the activator of the PI3K-AKT signal pathway (740 Y-P, 10, 20, and 30 μM, MCE, China). On days 1, 3, 5, and 7, proliferation of cells was detected by Cell Counting Kit‐8 (CCK-8, Beyotime, China). Cells in the working solution were incubated at 37°C for 2 h, and a Multiskan FC Microplate Reader (Thermo, USA) was used to measure the absorbance at 450 nm.

### Alkaline phosphatase activity assay and staining

After osteogenic induction with or without medical intervention for 1 week, alkaline phosphatase (ALP) staining and ALP activity assays were performed for ALP measurement. For ALP staining, the culture medium was removed, and adherent cells in the plates were washed gently with PBS, followed by fixation in 4% paraformaldehyde. In the dark and at room temperature, an ALP staining kit (Beyotime) was used to stain the cells. The percentage of positively stained areas was calculated using the Fiji 19.0 software (NIH, USA). Cellular ALP enzymatic activity was measured with an ALP assay kit (Beyotime, China). The activity was determined using para-nitrophenyl phosphate at 37°C for 10 min, then optical density was read and subsequently normalized against the total protein.

### Reverse transcription polymerase chain reaction (real-time PCR)

Total RNA isolated with TRIzol reagent (Takara, Japan) was transcribed into cDNA using Evo M-MLV RT Premix (Accurate Biology, China). SYBR^®^ Green Premix Pro Taq HS qPCR Kit (Accurate Biology) was used for real-time PCR. Furthermore, gene expression was calculated relative to that of β-actin. Table 1 shows the primer sequences.

**Table 1:** Sequences of primers.

Genes	Primers (5'-3')
*IL-6*	
Forward	ACAGCCACTCACCTCTTCAG
Reverse	GCCTCTTTGCTGCTTTCACA
*IL-1β*	
Forward	GCTGATGGCCCTAAACAGATG
Reverse	TGGTGGTCGGAGATTCGTAG
*TNF-α*	
Forward	TGAAAGCATGATCCGGGAC
Reverse	CCACGATCAGGAAGGAGAAG
*ALP*	
Forward	GGACCATTCCCACGTCTTCAC
Reverse	CCTTGTAGCCAGGCCCATTG
*COL1*	
Forward	AGACGAAGACATCCCACCAATC
Reverse	GATCACGTCATCGCACAACAC
*Runx2*	
Forward	GACGAGGCAAGAGTTTCACC
Reverse	GGTTCCCGAGGTCCATCTAC
*PI3K*	
Forward	TTATAGCAAGACTGTTAGCCCTC
Reverse	AGAGACGCAGACTAAAACCAG
*AKT*	
Forward	TCCGATTCACGTAGGGAAATG
Reverse	ATAGTGAGGTTGCATCTGGTG
*β-actin*	
Forward	GGACCATTCCCACGTCTTCAC
Reverse	CCTTGTAGCCAGGCCCATTG

### DNA library construction and high-throughput sequencing

The final cDNA library obtained from the qualified mRNA of DePDLSCs growing in osteogenic medium with or without LPS (10 μg/mL for 7 days) was run on the PromethION platform. Differential gene expression between the groups was analyzed using the DESeq2 R package (version 1.6.3). In addition, Gene Ontology (GO) enrichment analysis using GOseq R packages and Kyoto Encyclopedia of Genes and Genomes (KEGG) analysis using KOBAS software were performed for functional enrichment analysis. The cDNA library and full-length transcriptome sequencing procedures were implemented by Biomarker Technologies (China).

### Statistical analysis

All experiments were performed in triplicate. Data are reported as means±SE and were calculated using SPSS Statistics 20 (IBM, USA). One-way ANOVA corrected by Dunnett's *post hoc* test was applied for statistically different accessions, with a significance threshold set at 0.05. For quantitative analysis of transcript expression levels, Benjamini and Hochberg's approach was used to adjust the resulting P values to control the false discovery rate (FDR). Transcripts with an FDR <0.01 and fold-change ≥2 were defined as differentially expressed transcripts (DETs). Hypergeometric testing was used for GO functional annotation and pathway significance enrichment analysis.

## Results

### Isolation and identification of DePDLSC

**Figure 1:**
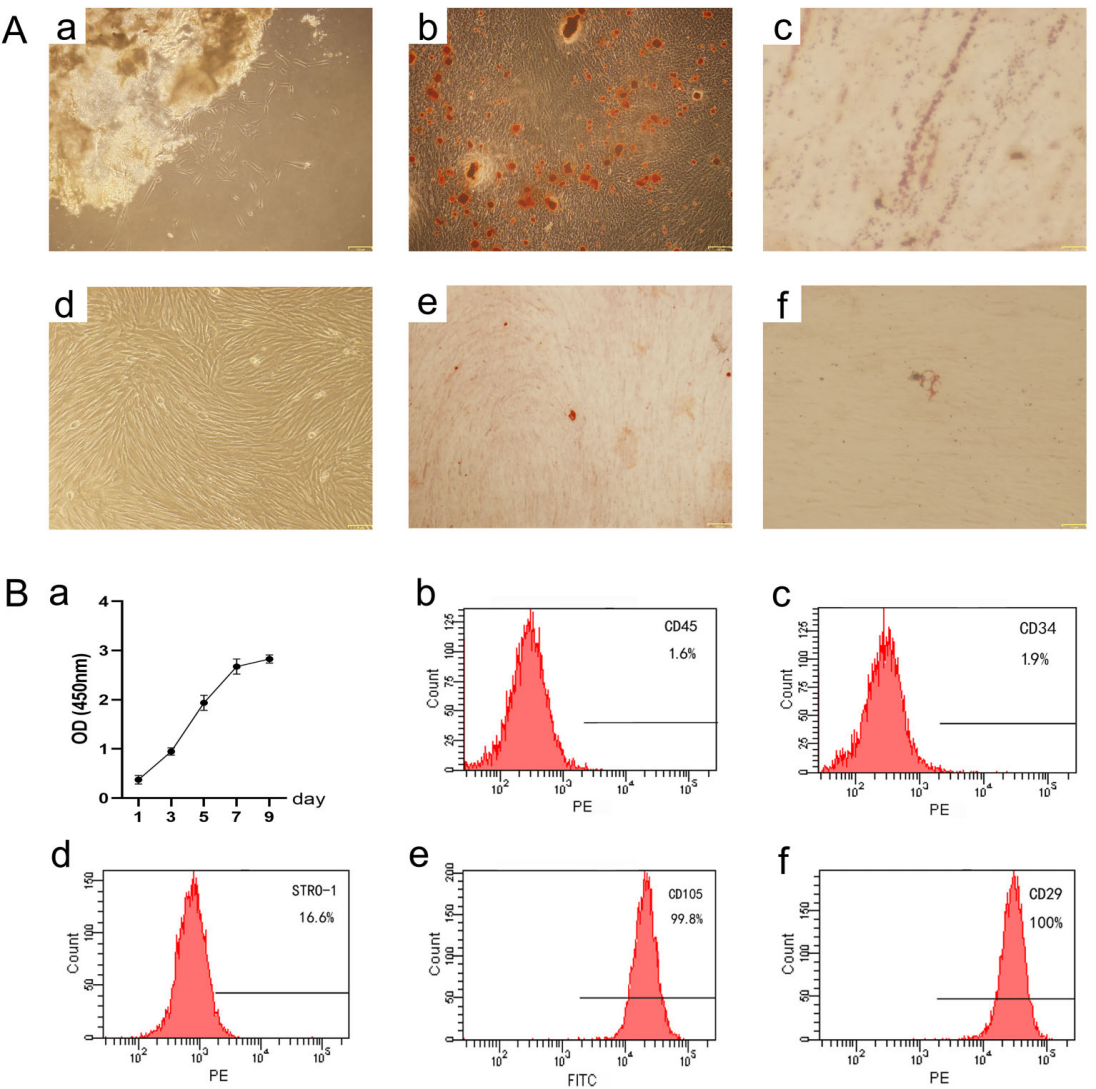
Isolation and identification of deciduous teeth periodontal ligament stem cells (DePDLSCs). **Aa**, Initial morphology of cells. Scale bar=150 μm. **Ab**, Alizarin Red S staining in the osteogenic group. Scale bar=150 μm. **Ac**, Oil red O staining in the adipogenic group. Scale bar=13 μm. **Ad**, Sub-cultured DePDLSCs. Scale bar=92 μm. **Ae**, Alizarin Red S staining in the control group. Scale bar=153 μm. **Af**, Oil red O staining in the control group. Scale bar=71 μm. **Ba**, Growth curve of DePDLSCs. Data are reported as means±SE. **Bb**-**Bf**, Flow cytometry results of DePDLSCs.

Primary cells were successfully obtained from the PDL tissue of the deciduous teeth and showed a spindle-shaped morphology similar to that of fibroblasts (Figure 1Aa and Ad). Cell growth accelerated on days 3 to 7, and then gradually retarded (Figure 1Ba). The flow cytometry data (Figure 1Bb-Bf) showed that the cultured cells positively expressed the mesenchymal stem cell markers (STRO-1, CD29, and CD105) and negatively expressed the hematopoietic markers (CD45 and CD34). In addition, mineralized nodules (Figure 1Ab) positively stained with alizarin red and lipid clusters (Figure 1Ac) positively stained with Oil Red significantly increased compared to the control group (Figure 1Ae and Af). The cultured cells were stem cells with multilineage differentiation ability.

### Impact of LPS on DePDLSC

**Figure 2:**
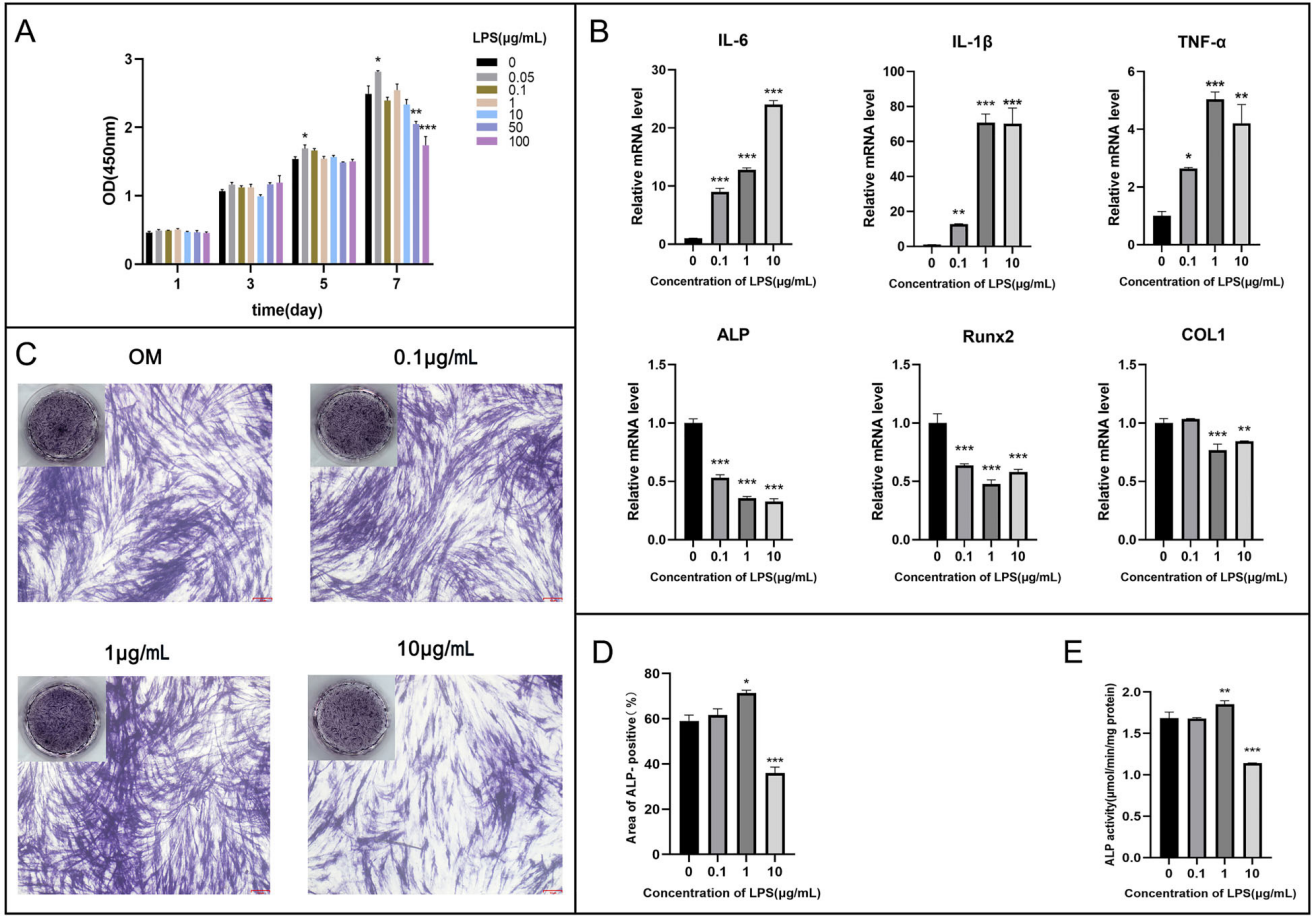
Effect of lipopolysaccharide (LPS) on deciduous teeth periodontal ligament stem cells (DePDLSCs). **A**, Proliferation of DePDLSCs upon LPS detected by CCK-8 assay. **B**, mRNA level of pro-inflammatory factors interleukin (*IL)-6*, *IL-1β,* tumor necrosis factor (*TNF*)*-α*, and osteogenic genes *ALP*, *Runx2*, and *COL1*. **C**, ALP staining of DePDLSCs captured by camera and microscope. Scale bar=153 μm. **D**, Semiquantitative results of ALP staining. **E**, Cellular ALP enzymatic activity results after LPS stimulation. *ALP*: alkaline phosphatase; *Runx2*: Runt-related transcription factor 2; *COL1*: Collagen type I; OM: osteogenic medium. Data are reported as means±SE. *P<0.05, **P<0.01, ***P<0.001 (ANOVA).

The CCK-8 assay data are shown in Figure 2A. On days 5 (F=5.636, P=0.001) and 7 (F=18.808, P<0.001), 0.05 μg/mL LPS significantly promoted cell proliferation. On day 7, cell proliferation was markedly impaired by 50 and 100 μg/mL LPS (F=18.808, P<0.001). Therefore, concentrations that showed no impairments (0.1-10 μg/mL LPS) were chosen for the subsequent experiments. Furthermore, real-time PCR results (Figure 2B) showed that proinflammatory factors *IL-1β* (F=867.752, P<0.001), *IL-6* (F=1192.978, P<0.001), and *TNF-α* (F=24.690, P<0.001) were significantly upregulated by 0.1-10 μg/mL LPS. In contrast, the expression of osteogenic genes *ALP* (F=406.883, P<0.001) and *Runx2* (F=74.797, P<0.001) notably decreased with 0.1-10 μg/mL LPS interference. *COL1* was inhibited by 1 μg/mL (P<0.001) and 10 μg/mL (P=0.001) LPS, except for 0.1 μg/mL LPS. ALP staining (Figure 2C and D) and ALP activity assay (Figure 2E) results revealed an increase in *ALP* expression with 1 μg/mL LPS incubation and a marked suppression with 10 μg/mL LPS intervention. The ALP levels between 0.1 μg/mL LPS group and control group were similar.

### Differentially expressed transcripts

**Figure 3:**
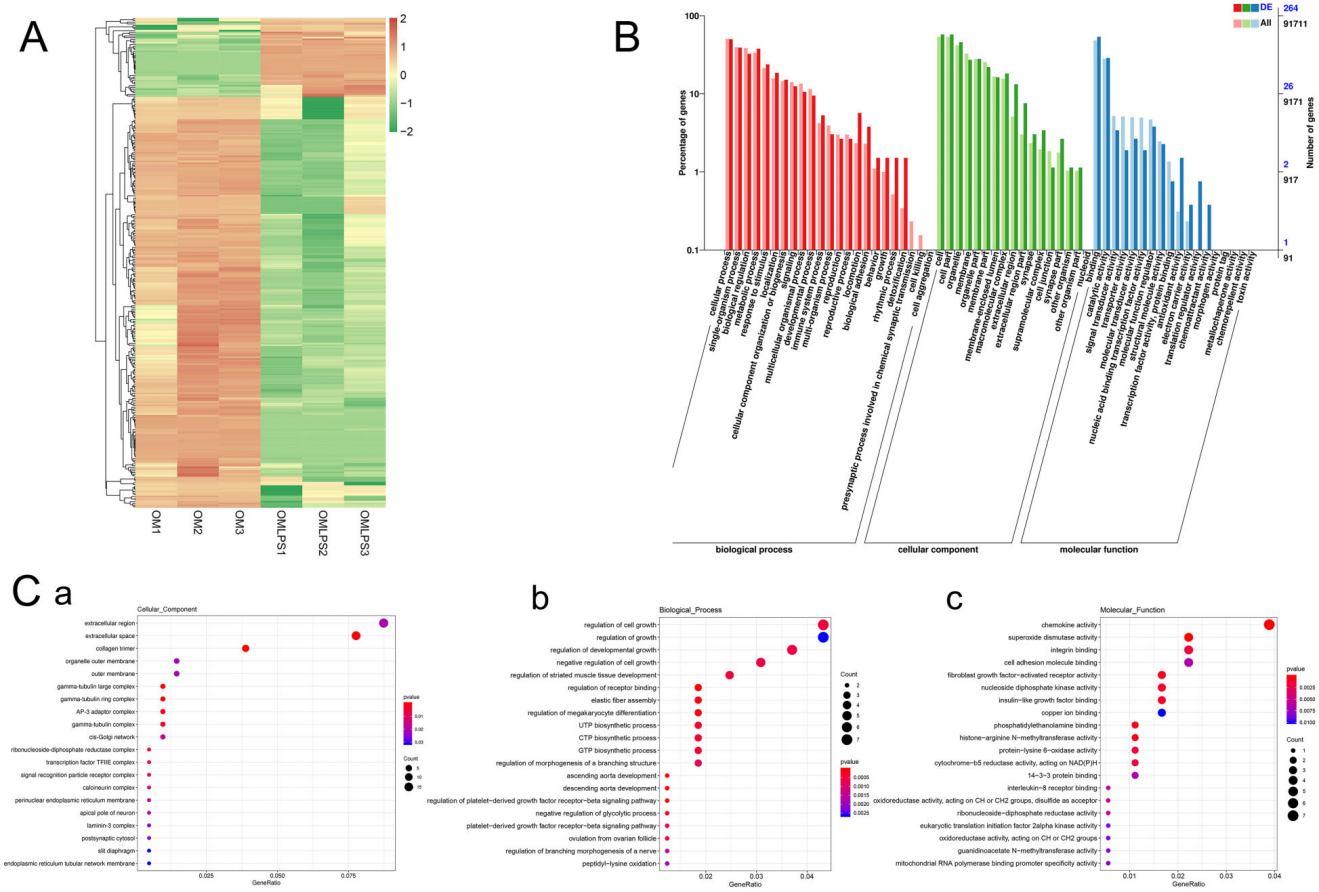
Data analysis of differentially expressed transcripts (DETs). **A**, Heatmap of DETs. **B**, Gene Ontology (GO) analysis of DETs. X-axis: GO classification. Y-axis: percentage of DETs under differentially expressed genes (DEGs) background and all genes background. **C**, DETs enrichment of GO secondary function. **Ca**, cellular component. **Cb**, biological process. **Cc**, molecular function.

DePDLSCs cultured in the OM (osteogenic medium) and OMLPS (osteogenic medium containing 10 μg/mL LPS) groups were harvested for transcriptome sequencing, with three biological replicates in each group. The filtered reads were called full-length sequences and aligned with the reference genome GRCh38 (*Homo sapiens*) using Minimap2 program (version 2.16). The sequence statistics are listed in Table 2. Clean reads aligned to the reference transcriptome were >96% in each sample, ensuring higher accuracy when conducting further comparisons. Heat maps of the DETs are shown in Figure 3A. According to the criteria, 285 DETs (46 of them were upregulated, 239 of them were downregulated) were detected. The top 10 most significantly upregulated transcripts were encoded by eukaryotic translation initiation factor 3 subunit H, C-X-C motif chemokine ligand 1 (*CXCL1*), transmembrane protein 222 (*TMEM222*), C-X-C motif chemokine ligand 8 (*CXCL8*), chromodomain Y-like (*CDYL*), adaptor related protein complex 3 subunit delta 1, *CXCL1*, insulin-like growth factor binding protein 7, C-X-C motif chemokine ligand 6 (*CXCL6*), and tetratricopeptide repeat domain 3 (*TTC3*). The top 10 downregulated transcripts were GNAS complex locus (*GNAS*), fibroblast growth factor receptor 1 (*FGFR1*), *TMEM222*, *TTC3*, pleckstrin homology like domain family A member 2, *CDYL*, *FGFR1*, cullin 4A (*CUL4A*), mitotic spindle organizing protein 2 B, and non-erythrocytic 1.

**Table 2:** Full-length sequence statistics.

Sample ID	Number of clean reads (except rRNA)	Number of full-length reads	Full-length percentage(%)	Mapped reads	Mapped rates(%)
OM-1	2681975	2367717	88.28%	2281841	96.42%
OM-2	2694547	2385714	88.54%	2298404	96.34%
OM-3	2830629	2523304	89.14%	2439282	96.67%
OMLPS-1	2488713	2214918	89.00%	2128778	96.11%
OMLPS-2	2554672	2282288	89.34%	2205930	96.65%
OMLPS-3	2853925	2564416	89.86%	2480468	96.73%

OM: osteogenic medium; OMLPS: osteogenic medium containing 10 μg/mL LPS. -1, -2, and -3 indicate that samples were tested in triplicate in each group.

### Annotation of DETs

GO annotation and KEGG analysis were performed to uncover the potential regulatory roles of DETs. The GO annotation system contained three main branches (biological processes, molecular functions, and cellular components) and additional secondary function branches. The DETs enriched in cellular components were related to “extracellular region” and “extracellular space” (Figure 3B and Ca). DETs annotated in biological processes were mainly associated with “biological adhesion”, “detoxification”, “locomotion”, and “rhythmic process” (Figure 3B and Cb). Moreover, “chemokine activity”, “integrin binding”, and “superoxide dismutase activity” were secondary branches in which DETs were mainly enriched in molecular functions (Figure 3B and Cc).

**Figure 4:**
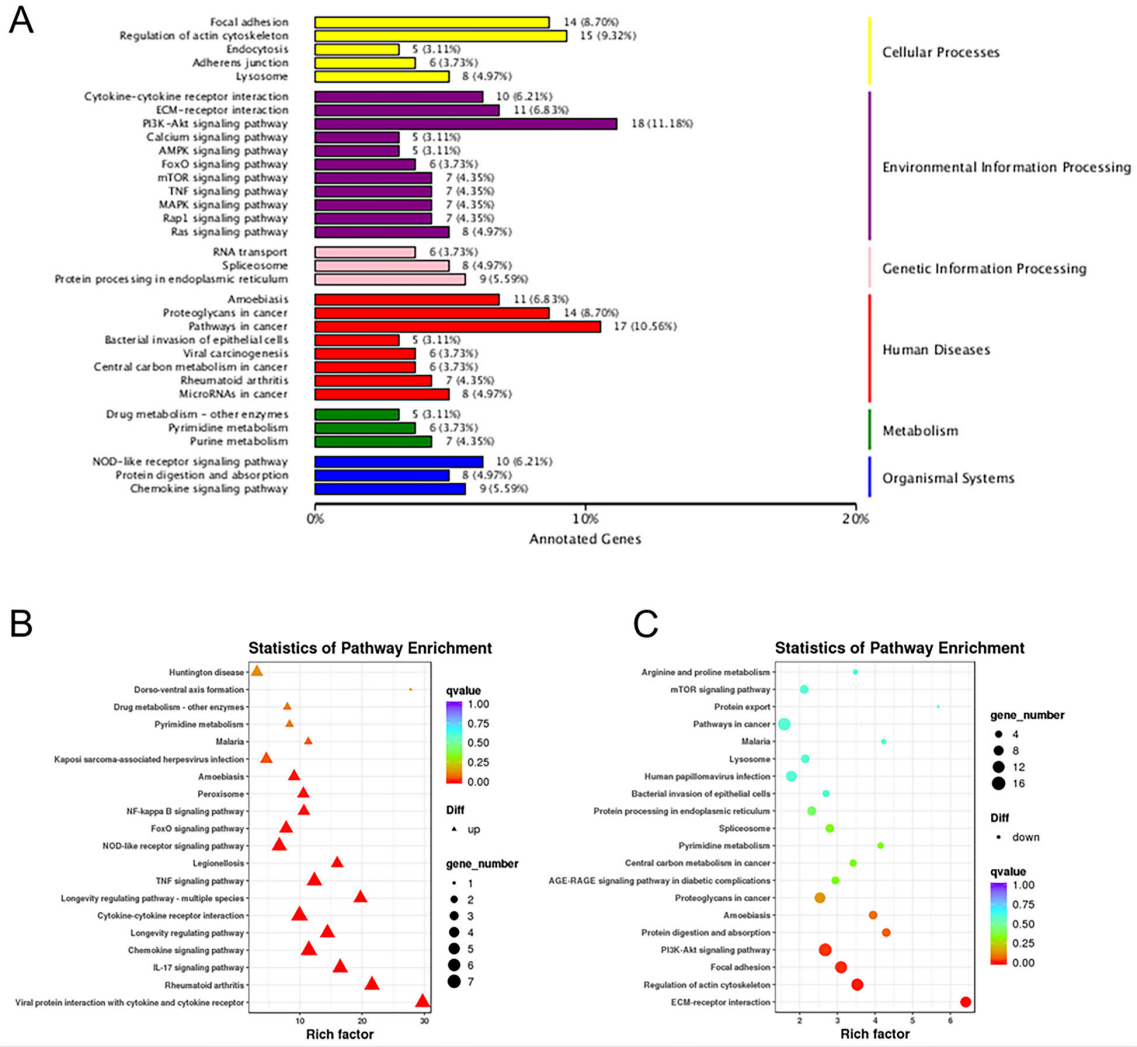
Kyoto Encyclopedia of Genes and Genomes (KEGG) analysis of differentially expressed transcripts (DETs). **A**, KEGG pathway classification. X-axis: number and percentages of DETs annotated in the pathway. Y-axis: name of the pathway. **B** and **C**, Pathway analysis of DETs. Rich factor: significance level of enrichment in this pathway. The Q value represents the reliability of the enrichment significance in the pathway.

According to the pathway types, the KEGG annotation results were categorized as shown in Figure 4A. Within the primary category “cellular process”, “environmental information processing”, and “human diseases” were strongly enriched. Furthermore, “regulation of actin cytoskeleton”, “PI3K-Akt signaling pathway”, and “pathways in cancers” were the secondary categories with which most DETs aligned. In the pathway significance enrichment analysis, 240 pathways were detected and ranked by Q value. Compared to the control group, LPS caused upregulation of enrichment in pathways: viral protein interaction with cytokine and cytokine receptor, rheumatoid arthritis, IL-17 signaling pathway, and chemokine signaling pathway (Figure 4B). In contrast, “ECM-receptor interaction”, “regulation of actin cytoskeleton”, “focal adhesion”, and “PI3K-Akt signaling pathway” were downregulated upon LPS stimulation (Figure 4C).

### Preliminary exploration of the effect of PI3K/AKT signaling pathway on osteogenesis ability of inflamed DePDLSCs

**Figure 5:**
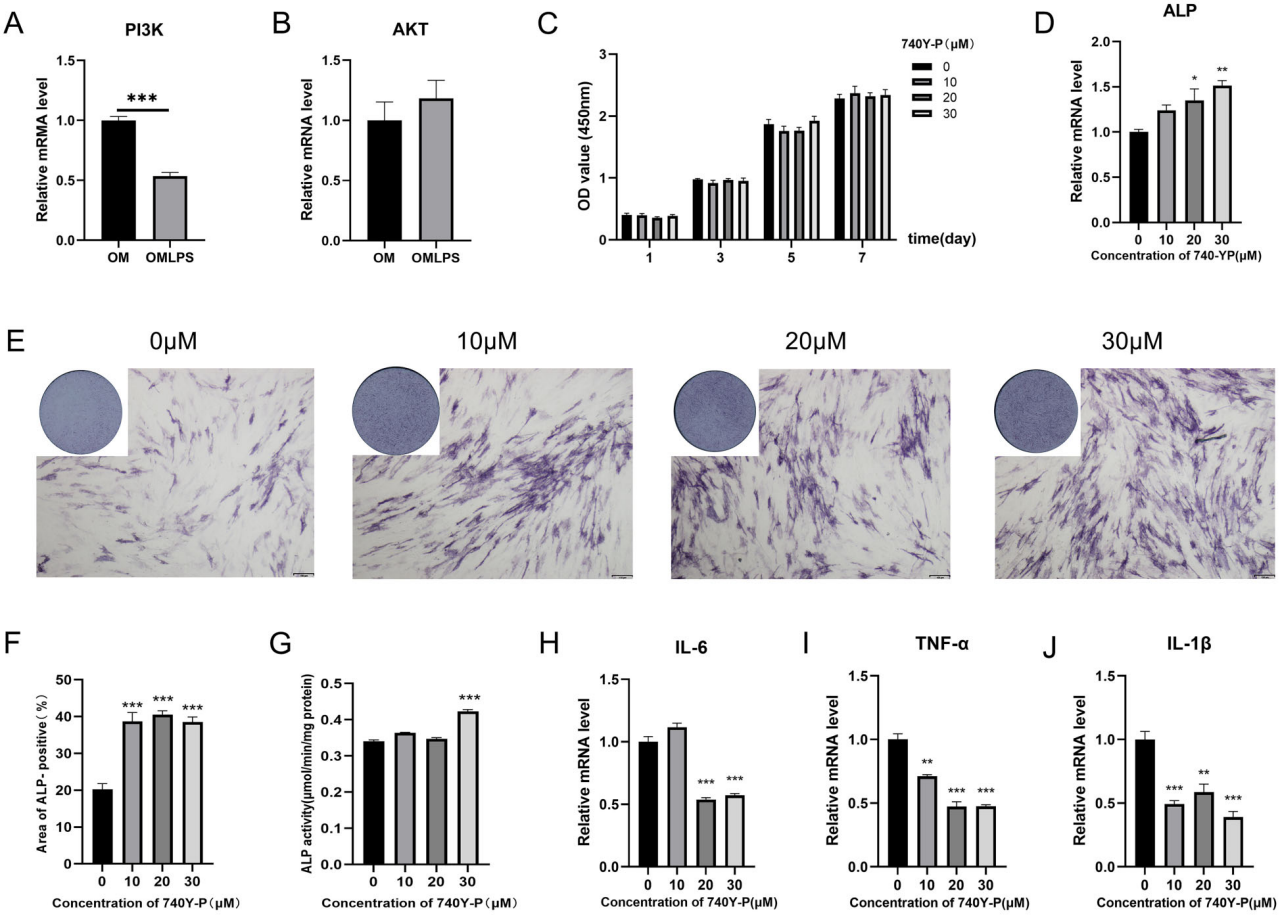
Effect of 740Y-P on deciduous teeth periodontal ligament stem cells (DePDLSCs). **A** and **B**, Real-time PCR validation of key genes in the PI3K-AKT signaling pathway. OM: osteogenic medium. **C**, 740Y-P showed no evident effect on cell proliferation. **D**, 20 and 30 μM 740Y-P increased alkaline phosphatase (*ALP*) gene expression in DePDLSCs. **E** and **F**, ALP staining of DePDLSCs with different concentrations of 740Y-P and semiquantitative results. Scale bar=150 μm. **G**, ALP activity results with 740Y-P stimulation. **H**-**J**, mRNA levels of the proinflammatory cytokines interleukin-6 (*IL-6*), tumor necrosis factor-α (*TNF-α*), and interleukin-1β (*IL-1β*). Data are reported as means±SE. *P<0.05, **P<0.01, ***P<0.001 (ANOVA).

Real-time PCR was used to preliminarily validate sequencing accuracy by testing the expression of key molecules in PI3K-Akt signaling pathway, PI3K and AKT. The results showed that LPS downregulated PI3K expression but not AKT expression (Figure 5A and B), consistent with the sequencing results. Furthermore, the effects of the PI3K/AKT agonist 740Y-P (10, 20, and 30 μM for 7 days) on proliferation activity, pro-inflammatory factors, and early osteogenic gene expression were measured using CCK-8 kit, real-time PCR, and ALP staining assay. The 10, 20, and 30 μM 740Y-P did not affect cell proliferation on day 7 (F=0.208, P=0.889, Figure 5C). However, 20 and 30 μM 740Y-P increased the mRNA level of *ALP* (Figure 5D), and ALP activity (Figure 5E-G) was significantly increased by 30 μM 740Y-P. Additionally, 20 and 30 μM 740Y-P significantly reduced the expression of *IL-6*, *IL-1β,* and *TNF-α* (Figure 5H-J).

## Discussion

The resorption of alveolar bone in deciduous teeth is much more severe than that in permanent teeth when inflammation affects the apical area. Moreover, apical periodontitis in deciduous teeth has a relatively high recurrence rate even post-RCT, and little is known about its underlying molecular biological mechanisms (16). The current study utilized an *in vitro* inflammation model. It employed high-throughput sequencing technology and found the essential function of PI3K/AKT signaling in the osteogenic differentiation of DePDLSCs under an inflammatory microenvironment induced by LPS.

The results of the assays demonstrated that proliferation of DePDLSCs was stimulated by low concentrations of LPS but suppressed by high concentrations. Owing to the complex interaction between LPS on cell proliferation and osteogenic differentiation, 0.1, 1, and 10 μg/mL LPS concentrations, which did not affect cell proliferation, were chosen for the osteogenesis tests to avoid cell numbers becoming a confounder. We observed that 10 μg/mL LPS suppressed the osteogenesis of cells while 0.1 μg/mL LPS stimulated it. Similar results have been reported in previous studies. When using homotypic LPS to stimulate PDLSCs, Xing et al. (17) determined the stimulatory impact of 0.05 μg/mL LPS, and Li et al. (18) discovered an inhibitory effect of 10 μg/mL LPS on the osteogenic differentiation ability of PDLSCs. A possible explanation for this phenomenon is the dynamic balance of the oral microbiota (17). Under physiological conditions, mesenchymal stem cells (MSC) benefit from low levels of LPS produced by dynamic and balanced bacterial communities. Contrarily, under pathological conditions, the excessive accumulation of certain bacteria causes them to become dominant pathogenic bacteria; the accumulation of LPS from dominant bacteria inhibits the differentiation ability of MSC. Additionally, a contradiction existed between *ALP* mRNA and protein expression, with 1 μg/mL LPS stimulation; this was probably due to posttranscriptional or protein translational changes.

Approximately 10 μg/mL LPS was chosen for modeling and subsequent high-throughput sequencing. In this study, 46 upregulated and 239 downregulated mRNAs were detected using sequencing technology. Notably, the upregulated transcripts (*CXCL1*, *CXCL8,* and *CXCL6*) are neutrophil chemoattractants, which can trigger inflammatory cascades and impair hard tissue (19-21). The upregulation of chemokines was similar to that in previous studies, where *CXCL6* and *CXCL8* of cells increased upon LPS stimulation (19). The significant downregulation of *ALP*, *COL1*, *GNAS*, and *FGFR1* transcripts upon LPS stimulation may indicate osteogenesis suppression in DePDLSCs. Mutations in *GNAS* are involved in pseudohypoparathyroidism and fibrous dysplasia of the bone (22). The critical roles of *FGFR*, *ALP*, and *COL1* in PDLSCs differentiation, cartilage osteogenesis, and bone metabolism have also been revealed in previous works (23-25).

GO analysis was performed on DETs. The annotation results showed that the top 10 upregulated transcripts were mainly located in the biomembrane, for instance, Golgi apparatus, lysosomes, and exosomes, indicating changes in protein synthesis in cells, as verified by KEGG annotation. The biological process also demonstrated that locomotion and biological adhesion, which can be used for wound tissue repair in PDLSCs (26), were changed by LPS.

According to KEGG analysis, the upregulated pathways in the OMLPS group were primarily related to the inflammatory reaction, indicating the inflammatory effect of LPS. Among the downregulated pathways, the “PI3K-AKT signaling pathway” has been reported to regulate osteogenesis and inflammation in stem cells, MSC, endothelial cells, and PDLSCs (27-29). However, this pathway has not yet been extensively studied in DePDLSCs. Therefore, the PI3K-AKT signaling pathway was chosen as a target to further explore the possible roles of DePDLSCs. First, the validation results for PI3K and AKT by qPCR were consistent with the sequencing data. A similar tendency was observed in other studies where LPS could downregulate the expression of phosphorylated (p) Akt, the activated form of AKT (30). Subsequently, 740Y-P, a specific PI3K activator that directly binds to the p85 N- and C-terminal SH2 domains of PI3K was used (31,32). Both the inhibition of inflammation and increase in ALP were detected after 740Y-P intervention. These results were consistent with those in some articles that through increasing the expression of PI3K or AKT, rutin, fucoidan, and mechanical stress could induce PDLSCs to differentiate into osteoblasts (33-35). Nevertheless, 740Y-P could also exacerbate *in vitro* inflammation in some studies, which is inconsistent with our findings (29). The activation environment of inflammation and the cell-type variation used in these studies may be related to the opposite effect of the same component regulating the PI3K signaling pathway. In addition, PI3K subtypes may have different functions in different diseases (36). Actually, other types of signaling pathways, such as NF-KB signaling pathway, can also regulate the osteogenesis of mesenchymal stem cell in LPS-induced condition (37). Nevertheless, because published research in which DePDLSCs were stimulated by LPS are very few, a parallel comparison is difficult.

In this study, we preliminarily identified that the PI3K-AKT signaling pathway was involved in the osteogenesis of DePDLSCs under inflammatory conditions; however, some limitations must be considered. First, PI3K-AKT signaling pathway contains various downstream molecules, and the specific intermediary or downstream transducers in the signaling cascade should be validated in further studies. Second, confirming the function of the PI3K-AKT signaling pathway in the late stages of DePDLSC osteogenesis is necessary to determine the best time for drug usage. Finally, rigorous *in vitro* and *in vivo* trials are essential before medication can be employed to treat apical periodontitis.

In conclusion, DePDLSCs proliferation and osteogenesis are affected by LPS in a biphasic manner. LPS (10 μg/mL) stimulated inflammation in DePDLSCs and decreased the expression of osteogenesis markers and the PI3K-AKT signaling pathway. However, the activator of PI3K-AKT signaling, 740Y-P, can rescue the secretion of inflammatory factors and ALP reduction. The current study provided new insights into the etiological probes for apical periodontitis and therapeutics.

## Data Availability

The datasets used and analyzed during the current study are available from the corresponding author on reasonable request.
